# A Treatment-Based Algorithm for the Management of Type-II SLAP Tears

**DOI:** 10.2174/1874325001812010282

**Published:** 2018-07-31

**Authors:** Adam M. Johannsen, John G. Costouros

**Affiliations:** Department of Orthopaedic Surgery, Stanford University, Stanford, CA, USA

**Keywords:** Biceps, Labrum, Shoulder, Tenodesis, Tenotomy, SLAP tear

## Abstract

**Background:**

The management of Type-II superior labral tears (SLAP) of the shoulder remains a controversial topic. Treatment ranges from non-operative management to surgical management including SLAP repair, biceps tenotomy, and biceps tenodesis. An optimal treatment algorithm has yet to reach universal acceptance.

**Objective:**

The goal of this paper was to provide a treatment algorithm for the management of Type-II SLAP tears based on current literature and expert opinion.

**Method:**

Current literature was reviewed and expert opinion was reported to develop a comprehensive treatment protocol for patients based on age, activity level, and pathology.

**Results:**

Operative management of type-II SLAP tears yields good to excellent outcomes when proper indications are followed. Biceps tenodesis may produce more reliable pain relief and functional improvement when compared to primary SLAP repair in patients over the age of 40.

**Conclusion:**

When non-operative management of Type-II SLAP tears fails, operative management yields good to excellent outcomes in most patients. Primary SLAP repair should be performed in patients under the age of 40 with no evidence of proximal biceps pathology, while biceps tenodesis can provide consistent pain relief and return to activity in patients over the age of 40 or with significant proximal biceps pathology. Tenotomy should be reserved for elderly or low demand individuals.

## 1 INTRODUCTION

The management of type-II superior labral tears (SLAP) remains a diagnostic and therapeutic challenge. After exhausting non-operative treatment including physical therapy, activity modification, and anti-inflammatory medications, surgical management may be considered. Surgical treatment options range from arthroscopic superior labral debridement, labral repair, and biceps tenotomy or tenodesis with our without superior labral repair. Treatment decisions have historically been based on patient age, activity level, and concomitant pathology. However, outcomes studies have displayed mixed results, and a clear treatment algorithm has yet to be defined.

Previous literature has stated the importance of maintaining the superior labral-biceps complex to preserve glenohumeral stability [1-3]. In the circle concept, both the superior labrum and long head of biceps tendon have been suggested to act as a superior restraint to humeral head translation. Both clinical and biomechanical studies have demonstrated that biceps tenotomy and tenodesis result in greater humeral head translation compared to SLAP repair [1-3]. However, SLAP repair has risks of failure including the failure of healing, concomitant biceps tendonitis, stiffening of the capsulolabral complex due to over-tensioning of the labrum and glenohumeral ligaments, residual instability, hardware complication including rotator cuff irritation, and perhaps erroneous treatment of labral variants [4]. Further, clinical outcome data has not consistently proven the significant functional benefit of arthroscopic SLAP repair over tenodesis [4-6].

Several recent studies have contributed to our understanding of outcomes following surgical treatment of SLAP lesions. A retrospective cohort study demonstrated improved outcomes in the biceps tenodesis group compared to the SLAP repair group [5]. Chalmers *et al*. displayed in a retrospective non-randomized study that biceps tenodesis and superior labral repair performed similarly [4]. However, in this study, a subset of patients had both biceps tenodesis and labral repair performed. These patients had significantly worse outcomes than either procedure in isolation. Both of these studies were retrospective in nature and lacked a clear control group, and, therefore, their results should be interpreted with caution. A recently published randomized controlled trial was conducted comparing sham surgery to labral repair and biceps tenodesis [6]. In this blinded study, there were no clinical differences between labral repair or biceps tenodesis as compared to sham surgery at 2 years of follow-up. As a result of this inconsistency in the peer-reviewed literature, there continues to be a diversity of opinion in how to treat these injuries amongst surgeons.

In the absence of clear consensus in the peer-reviewed literature, surgeons must rely on a summary of existing studies in the context of personal experience. The following review presents a combination of clinical experience in conjunction with the current data available in the peer-reviewed literature for the treatment of type-II superior labral tears of the shoulder.

## 2 METHODS

This study developed an evidence based treatment algorithm for type-II SLAP tears based on current literature review and expert opinion. Current and past literature were reviewed and the most critical papers are referenced in the text. As this study did not directly involve human specimens, internal review board approval was not needed and human and animal rights were not directly involved.

## 3 AUTHORS PREFERRED METHOD OF TREATMENT

**Fig. (1):**
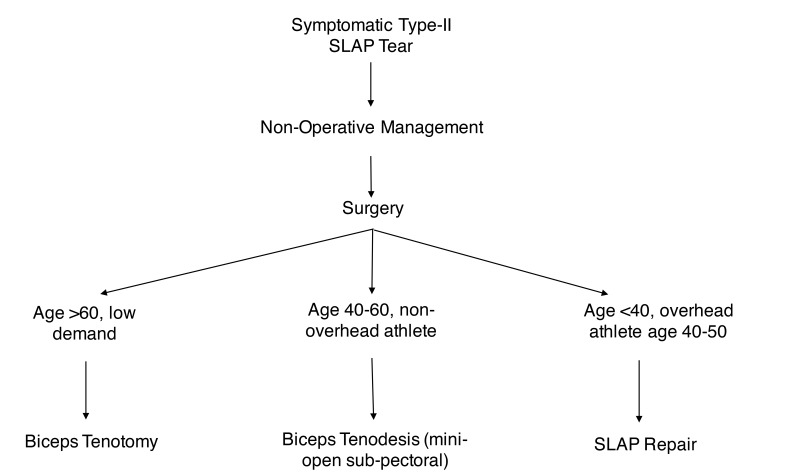
This image displays the authors preferred treatment algorithm for the management of Type-II SLAP tears. Note that these are general guidelines and not strict criterion.

The treatment for type-II SLAP tears depends on several factors including the site and severity of injury, involvement of a diseased proximal biceps tendon, patient age, activity level, and concomitant injuries (Fig. **1**). In all patients, non-surgical treatment should be exhausted including physical therapy, activity modification, and Non-Steroidal Anti-Inflammatory Medications (NSAID’s). For patients that fail 6 weeks to 3 months of non-operative management, selective injections including the biceps tendon sheath or intra-articular injections can be utilized for diagnostic or therapeutic purposes. We have not found intra-articular injection with steroid or newer, commercially available biologic agents such as platelet-rich plasma to be effective in a consistent or durable fashion. Use of these agents in combination with a local anesthetic may provide useful diagnostic information [7].

For active patients and those with persistent daily symptoms, surgical management should be considered when non-surgical management fails. The sections below highlight different patient types and the authors approach in each scenario. The age limit of 40 is a loose indicator based on prior literature and the personal experience of the senior author, but the patient’s physiologic age should be considered [8]. However, it is important to note that the surgeon must understand the patient’s goals and activity level prior to committing to a specific surgical plan.

## 4 YOUNG PATIENT (<40), ACTIVE, OVERHEAD ATHLETE: SLAP REPAIR

In the young, active patient population, primary SLAP repair is the preferred treatment strategy. These patients rarely have full-thickness rotator cuff tears. If a partial rotator cuff tear is encountered in addition to the SLAP tear, rotator cuff debridement is the main treatment that should be considered in order to preserve overhead throwing ability at a high level. During the diagnostic arthroscopy, the biceps tendon is arthroscopically visualized as it is retracted into the joint and assessed for pathologic changes or instability associated with disruption of the biceps pulley mechanism. If significant pathologic changes are noted including biceps subluxation, disruption of the biceps pulley complex, significant interstitial tearing, or profound tenosynotivitis, surgery is converted to an open subpectoral biceps tenodesis [9], and additional stabilization of the SLAP lesion is performed only if an extensile associated labral tear is encountered extending posteroinferiorly or anteroinferiorly in addition to the superior labral tear beyond the 11-1 0’clock position. Anterosuperior stabilization should be avoided in the context of normal labral variants (sublabral foramen, Buford complex). It is possible that some of the reported poor results of combined biceps tenodesis and superior labral repair are the result of repair of some of these normal, non-pathological labral variants or over-aggressive tensioning of the superior labral complex [4].

Arthroscopic SLAP repair is performed in the beach-chair position, utilizing the standard anterior and posterior viewing portals. In addition, an accessory high anterior portal or trans-cuff portal may be used is some cases for access to the superior glenoid [10]. In most cases, a single knotless anchor at the 12-0’clock position is sufficient for stabilization of the superior labral tear and in order to avoid overly aggressive tensioning of the superior labral complex. Prior to labral repair, the superior glenoid should be debrided to create a bleeding bony bed in order to enhance subsequent labral healing. In general, knotted anchors are avoided in this region of the labrum as these may irritate the undersurface of the rotator cuff. The authors prefer a lasso technique where a single No. 2 fiberwire (Arthrex; Naples FL) is passed around the torn biceps anchor at the 12 0’clock position. This is then fixed in place with a knotless suture anchor [11]. Furthermore, special care should be taken to avoid unnecessary repair of the anterosuperior labrum as this may lead to profound postoperative stiffness, usually in external rotation.

The postoperative physical therapy program following SLAP repair involves the typical three-phase protocol: protection phase, active phase, and strengthening phase. A sling is worn at all times for the first 4 weeks, except under guided physical therapy. For superior labral repairs in particular, initiation of immediate passive range of motion exercises, especially In external rotation is critical. Following the first 4 weeks, the sling may be discontinued and active range of motion exercises of the shoulder can be initiated. Strengthening of the rotator cuff begins at 12 weeks postoperatively and a full return to activities is allowed between 14-20 weeks based on clinical evaluation and performance in a supervised throwing program with a physical therapist.

## 5 PATIENT AGE >40, MILD TO MODERATE ACTIVITY, OR MANUAL LABOR: BICEPS TENODESIS

In the middle aged patient population, SLAP tears are often accompanied with proximal biceps pathology. In these patients, it is important to note pre-operatively whether they have anterior shoulder pain, tenderness to palpation at the bicipital groove, or positive provocative maneuvers for proximal biceps pathology including a positive Speed’s or Yergason’s test. Additional pathology such as rotator cuff tears or subacromial impingement may be identified and need to be addressed at the time of surgery. Diagnostic injections with ultrasound guidance can be useful in determining the actual source of shoulder pain [12]. A recent randomized controlled trial compared tenotomy, tenodesis, and simple debridement following rotator cuff repair for patients with both SLAP lesions and rotator cuff tears [13]. This study demonstrated clinical improvement in all groups, but improved supination strength and fewer popeye deformities in the biceps tenodesis group as compared to the tenotomy group. Therefore, we prefer arthroscopically-assisted mini-open subpectoral biceps tenodesis in the middle-aged patient with moderate degrees of activity needs with or without concomitant rotator cuff pathology.

Biceps tenodesis surgery is performed in the beach-chair position with an arm holder (Spider, Smith & Nephew; Andover, MA). Surgery is started with a comprehensive diagnostic arthroscopy of the shoulder and the treatment of any additional pathology. The proximal biceps tendon is cut at the superior labrum. Following completion of the arthroscopy, the arm is externally rotated and the subpectoral tenodesis is performed. A 2-3cm vertical incision is centered over the biceps tendon in the axilla, at the level of the inferior border of the pectoralis major tendon. While the pectoralis major tendon is retracted superiorly, the long head of the biceps tendon is identified and pulled out through the incision. The tenodesis site is identified in line with the bicipital groove at the level of the inferior margin of the pectoralis tendon and the cortical bone is gently debrided with a rasp or osteotome to stimulate a bleeding response. In general, the musculocutaneous junction of the long head of the biceps lies at the inferior border of the pectoralis major tendon; this serves as a useful reference for determining the appropriate position of the tenodesis. We prefer double loaded cortical bone suture anchors which allow for stable initial fixation. One limb of each strand is fixed to the tendon using locking stitches. The free limb is then used as the post to tension the tendon to the bone, and each strand is then tied individually.

For patients who undergo isolated biceps tenodesis without superior labral repair, immediate active and passive range of motion of the shoulder is allowed as well as immediate rotator cuff strengthening. Immediate active and passive range of motion of the elbow is also allowed. However, elbow flexion against any resistance is not allowed for four weeks postoperatively. The sling is primarily used for the first four weeks following surgery to protect against resisted active elbow flexion. Further, activity restrictions are dictated by any associated rotator cuff repair or other procedures which may require more extensive restrictions. If the patient is doing well at the three-month post-operative visit, they are allowed to return to full activities at their discretion.

## 6 ELDERLY PATIENT, LOW DEMAND: BICEPS TENOTOMY

In the elderly, low demand patient biceps tenotomy is preferred and produces reliable results. This technique is technically easy, minimizes the morbidity of an additional incision, and allows immediate passive and active range of motion of the shoulder. Similar to the tenodesis protocol, elbow flexion against resistance is not allowed for the first four weeks postoperatively. However, full shoulder and elbow range of motion is allowed immediately. Rotator cuff strengthening may also be initiated immediately following surgery. The functional impact of biceps tenotomy remains controversial, as do the factors that are predictive of the development of a ‘popeye’ deformity following surgery [14]. The popeye deformity can occur in approximately 10-25% of patients. Therefore, in patients with large cosmetic concerns, tenotomy should be avoided. Arm cramping is also a risk following tenotomy, but this typically self-limited by 3-4 months post-operatively if it occurs and rarely is a major long-term complaint.

Surgery is performed in the beach-chair position in a manner similar to the biceps tenodesis protocol described above. The biceps tendon is released off of the labral insertion, and variable degrees of retraction can be observed following tenotomy depending on the presence or absence of adhesions more distally along the course of the tendon. Following surgery, the patient does not have any restrictions to active or passive shoulder or elbow range of motion. A sling is used for comfort only and the only restriction is that elbow flexion against resistance is prohibited for four weeks following surgery. Again, if biceps tenotomy is performed in conjuction with other procedures such as rotator cuff repair, additional restrictions are required.

## CONCLUSION

Management of Type-II SLAP tears remains a diagnostic and therapeutic challenge, especially in the context of lack of consensus in the literature. Surgical management includes labral repair, biceps tenodesis, biceps tenotomy, or a combination. Clinical decision making should be based on patient age, desired activity levels, the degree of participation in overhead sports, and the presence or absence of other associated pathology. These variables should be considered carefully as the surgeon and patient develop the ideal surgical treatment plan after conservative measures have failed.

Erickson *et al*. showed that age >40 is a risk factor for failure and post-operative stiffness in SLAP repair [8], and that biceps tenodesis has good to excellent outcomes in the age >40 group. Therefore, our general threshold for treatment with biceps tenodesis is age 40. Chalmers *et al*. displayed in a non-randomized study that combined biceps tenodesis and SLAP repair are not recommended due to poorer outcomes than either procedure in isolation [4]. Therefore, we do not routinely perform or recommend combined procedures at our institution. In addition, SLAP repairs benefit from the initiation of early supervised physical therapy in order to minimize the risk of stiffness following surgery, particularly in external rotation.

## LIST OF ABBREVIATIONS

SLAP: Superior Labral Tear from Anterior to Posterior
NSAID’s: Non-Steroidal Anti-Inflammatory Drugs
